# The Effect of Recycled Geogrid Fibres on Asphalt Concrete Performance: A Case Study from Poland

**DOI:** 10.3390/ma17235923

**Published:** 2024-12-03

**Authors:** Grzegorz Mazurek, Przemysław Buczyński, Artur Kowalczyk

**Affiliations:** Department of Civil Engineering and Architecture, Kielce University of Technology, Al. Tysiąclecia Państwa Polskiego 7, 25-314 Kielce, Poland; p.buczynski@tu.kielce.pl (P.B.); akowalczyk@tu.kielce.pl (A.K.)

**Keywords:** geogrid recycling, reclaimed asphalt pavement, composition optimisation, reclaimed asphalt pavement, design of experiment, survey

## Abstract

This study articulates findings from research pertaining to the utilisation of recycled geogrid in asphalt concrete. The issue of contamination of reclaimed waste with geosynthetic materials persists as a significant concern that warrants attention. In Poland, the allowable quantity of geogrid contaminants within the mineral–asphalt composition is 0.1% *w*/*w*. The preliminary evaluation of the validity of the research topic was conducted based on an analysis of correspondence and survey outcomes. The fundamental material research was executed employing elements of experimental design theory. The experimental domain considered two qualitative factors: the type of bituminous mixture for the binding layer (AC16W and AC22W) and the type of geogrid material (glass, carbon), in addition to two quantitative factors: the length of the recycled geogrid fibres ranging from 1 cm to 5 cm, and the quantity of recycled geogrid fibres varying from 0.2% to 1%. A generalised linear model was employed for the analysis, enabling the consideration of dependent qualitative factors in forecasting characteristics. The qualitative evaluation of the resultant solution was conducted using multi-criteria optimisation via the Harrington function. Consequently, recycled carbon mesh fibres demonstrated a notably positive impact, enhancing the material’s quality by 22%. Regarding glass mesh, the fibre content should not exceed 0.2% in the AC22W mixture, whereas it can be increased to 1% in the AC16W mixture. Comparing all evaluated mixtures, it was ascertained that surpassing the allowable contamination limit of 0.1% in geogrid form does not result in a significant reduction in the quality of asphalt concrete compared to the reference mix.

## 1. Introduction

More than 2.1 billion tonnes of waste are produced annually in the European Union. The amount of waste and its management methods vary considerably between EU countries [1]. According to the data, 36% of the waste is attributed to Construction and Demolition Waste (CDW) such as concrete, bricks, tiles, ceramics, wood, glass, metals, and plastic. The second largest category, comprising 26% of the waste, is Mining and Quarrying Waste. Other categories include Industrial Waste (10%), Municipal Solid Waste (10%), Agricultural and Forestry Waste (8%), Waste from Services and Commerce (5%), and Hazardous Waste (4%). Consequently, waste generated from construction activities represents the largest portion of waste production, inclusive of polymer waste. In the German economy, the amount of recovered waste in the form of asphalt waste in 2022 was 14,800,000 tonnes, of which up to 87% was effectively reused in the production of asphalt mixtures. In 2022 in Poland, according to a study [2], asphalt waste was mostly used to reinforce road shoulders, which is not, according to the authors’ assessment, the best way to use the potential of asphalt waste. Poland, as one of the countries of the European Union, has to adapt to various environmental and climate protection initiatives (Closed loop economy—GOZ and the European Green Deal) [3,4]. The authors of the report [4] highlight that the European Union is developing proposals to support the transition to a more efficient circular economy, where resources such as asphalt waste are used more sustainably. The risks posed by the presence of recycled fibres in the asphalt RAP generates the need to try to assess the impact of contamination by geogrid fragments that end up in the asphalt mixture on the properties of asphalt concrete and the possibility of its reuse in the production of new asphalt layers, which fits in with the tenets of sustainable road construction. It should be emphasized that geogrids, composed of glass fibres or polycarbonates, are characterized as stable and chemically inert substances. Under typical environmental conditions, they do not release harmful chemicals and do not decompose into toxic by-products. Nonetheless, the provisions set forth in the harmonized European standard EN 13108-8 identify RAP contamination as involving polymeric materials. Within the context of road engineering, geogrids are regarded as pollutants due to their potential for incorrect degradation or improper disposal, which can result in microplastic pollution. Consequently, their uncontrolled presence in RAP inhibits the further recycling of this material and should therefore be addressed as a contamination issue.

Roads shall be designed and constructed with a view to ensuring that they are suitable for the volume of traffic to be carried and for the adverse effects of weather conditions. Asphalt pavements, which can be classified as either flexible or semi-rigid structures [5,6], represent the dominant road construction technology in Poland and worldwide [7]. The total production of mineral and asphalt mixtures in 2022 in Europe, according to the EAPA report [8], was 279.4 million tonnes. In modern technology, the procedure of fibre reinforcement of pavement materials has been used for many years [9]. The first applications of fibres in mineral and asphalt mixtures took place in the 1920s [10]. There are many types of fibres that can be added to an asphalt mixture [9,11]. Fibres are stranded structures that can be classified by raw material category as natural fibres, unnatural fibres, and waste fibres. Glass and carbon fibres from which geogrids used for asphalt layers are made [12]. Recycling these fibres from asphalt pavement can be a cost-effective and efficient way to recycle waste glass and carbon geogrids, whereby a reduction in the cost of producing MMA reinforced with, for example, new dispersed fibre is achievable. The authors of the publication [13] note that changing macroeconomic conditions are forcing us all to take action to reduce the environmental impact of people, organisations, processes, or products. In the pursuit of environmentally benign solutions, a plethora of scientific literature exists that has investigated the impact of diverse fibre types on the quality of mineral and asphalt mixtures [11]. The literature review [11] identifies numerous implementations within the realm of geogrid applications. These geogrids are integrated as materials that enhance the mechanical properties of MMA, such as resistance to permanent deformation and fatigue life [14]. The deployment of geogrids is shown to positively impact the characteristics of WMA blends, inclusive of the ITS parameter [15]. In the study [16] utilising rock lattices, the authors report a 13% enhancement in the stiffness of the mineral–asphalt composite, as per Marshall’s evaluation, with the application of 0.1% glass fibres. Conversely, research conducted with various fibre types (including nylon, polyester, and polypropylene) determined the optimal fibre content to be 1% [17]. Nevertheless, existing studies, e.g., [18], have not evidenced any alterations in asphalt properties resulting from geogrid integration, thereby corroborating its minimal reactivity with asphalt.

The field of road construction in Poland is undergoing a period of rapid technological advancement. Technological progress can be defined as a process that ultimately results in the development of novel solutions. Simultaneously with the advancement of technology, there is an increasing focus on the recycling of materials, including those utilised in road construction. The transition of industrial production to an economic system that aims to optimise the utilisation of resources through reuse and recycling is becoming increasingly prevalent [19]. The objective of a closed-loop economy is to establish a system in which waste is regarded as a secondary resource. The circulation of products and materials within the economy is a key factor in achieving sustainability, with the potential to significantly reduce the environmental impact of construction. An illustrative example of a malleable waste material employed in road construction is asphalt waste, which is generated through the process of milling rehabilitated asphalt pavements [8]. A significant factor in evaluating the quality of asphalt pavement decomposition is the presence of extraneous materials, particularly those belonging to Group Two, which encompasses a range of materials including plastics, synthetic substances, and wood [20]. The issue of contamination of asphalt de-compacts with geosynthetic fibres is becoming increasingly prevalent. Over the past few years, it has become increasingly common to find a layer of glass or carbon geonets in the pavement structure when milling rehabilitated pavements. Contamination of the asphalt waste with the fragmented geogrid occurred during the milling process for this type of construction. The current state of affairs is limited, given the regulations in force in Poland on the design of bituminous concrete, due to the exceeding of the permissible content of foreign materials in asphalt granulate (<0.1%) [20,21,22], and the reuse of asphalt waste contaminated with recycled geogrid fibres. As a result, there has been an increase in the amount of geogrid and asphalt waste, as well as asphalt destruction. The question of the effect of reinforcing fibres on the durability of mineral asphalt mixes is frequently raised in the scientific literature, but as aforementioned [11], in relation to newly produced fibres. Currently, fragments of geogrids in asphalt decompaction are counted as asphalt decompaction contaminants. It should be noted that the amount of new macro glass fibres [23] and other fibres dispersed in mineral–asphalt mixtures, according to an analysis of the technical literature, significantly exceeds the permissible standard of synthetic impurities according to the requirements of WT-2/2014 [22]. Depending on the type of fibre and the intended use, the dosage of fibres in asphalt mixes varies. New glass and carbon fibres are mostly added to MMA in the range of between 0.2% and more than 4% of the total weight of the mixture and are not seen as a contaminant [11]. Dispersed glass and carbon fibres are considered as a full-fledged additive to improve the physical, mechanical, and fatigue properties of mineral–asphalt mixtures [11,23,24]. Although fibre reinforcement in mineral–asphalt mixtures is considered to be incidental [25], many researchers believe that fibres act as a bridge in cracks, absorbing, redistributing, and transferring stresses, thereby preventing or delaying crack propagation [23,26,27]. According to studies [17,28,29,30], improvements in the tensile strength and rutting resistance of mineral–asphalt mixtures treated with dispersed fibres were also found. According to the authors, further research is recommended on the compatibility of fibres and asphalt mixtures, sourcing them in an economically viable and sustainable manner [11]. In a study carried out in Germany [31], it has been proven that glass–carbon geonets can be implemented into road pavement structures without any problems, and that they also behave very well after their service life during pavement milling. As far as the recycling of the geonet is concerned, the study found that the shredded product can be fully reusable in a mineral and asphalt mixture. Furthermore, according to the report [31] based on the rutting resistance tests carried out, it was shown that the fragments of a particular S&P Glass/Carbonphalt G geogrid do not adversely affect the properties of the asphalt concrete. It was concluded that the fragmented residues of the geogrid reinforcement can, without problems and in accordance with the recommendations of German laws and regulations on waste management and product recycling, be used for rational recycling, e.g., in mineral–asphalt mixtures [32]. In contrast, in the area of Polish experience, on the basis of the PSWNA research paper entitled “Studies on the use of reclaimed asphalt pavement in Poland”, it can be stated that although reclaimed asphalt pavement is an extremely valuable raw material that can be successfully reused in the construction of asphalt layers in Poland, Poland does not have a sufficient volume of this raw material [2]. The study carried out by the PSWNA is an attempt to answer, among other things, questions about obstacles to the use of the asphalt RAP, the potential of companies, incentives in use, demand, etc. With the geogrid fibre content in the RAP already as low as 0.1%, according to the requirements of WT-2/2014, such RAP cannot be reused for the production of MMA. From the analysis of the PSWNA study [2], on the other hand, up to 80% of Polish construction companies declare that they use asphalt waste in their operations. Manufacturers of mineral and asphalt mixtures usually specify that the share of asphalt waste in the composition of asphalt concrete is 20% [2].

In view of the discussion that followed from the literature review, one of the main research objectives of this study was the possibility of implementing geonet fibres found in the waste at >0.1% *w*/*w* with different lengths into asphalt concrete. It should be added that knowledge of the behaviour of the asphalt concrete containing the waste material, which carries the geonet fibres, will allow a more rational use of the waste material. A key element of this study was to map the state and distribution of the geonet in the mineral–asphalt mixture as close as possible to real conditions. As the subject matter is not recognised in Poland, the research procedure was enriched with a survey.

## 2. Survey

**Figure 1:**
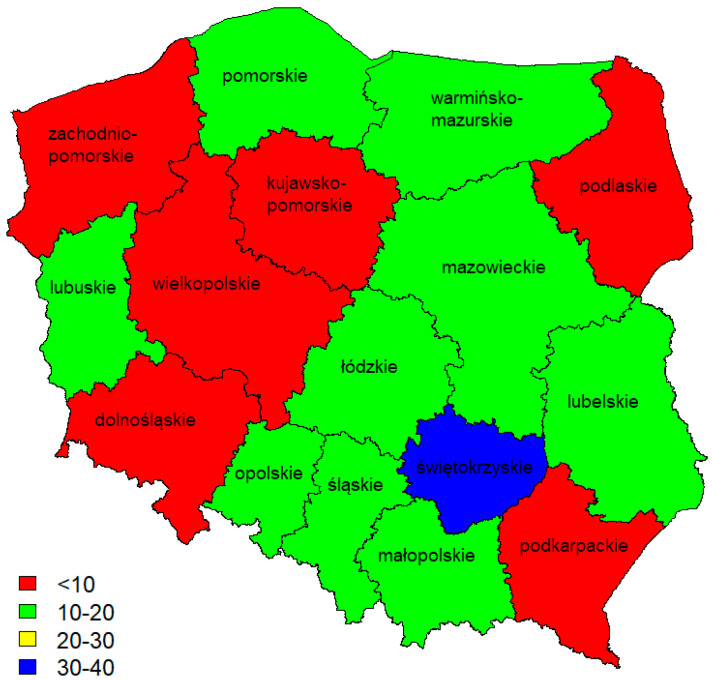
Number of respondents by province.

According to the aforementioned WT-2/2014 recommendations in Poland, the content of impurities in the form of plastic materials in the reclaimed asphalt pavement (RAP) should be <0.1% (category F1 according to [20]). However, under practical conditions, it is very difficult to effectively clean the asphalt granules of this type of contamination. This raises the question of whether every contaminant in the form of geogrid elements must absolutely impair the properties of mineral–asphalt mixtures. Furthermore, it is also important to know what scale of contamination in the form of geogrid fibres is encountered by manufacturers of mineral and asphalt mixtures containing asphalt waste. Responses were obtained from a survey conducted in 2023, which assessed expectations and perceptions regarding the presence of contaminants in the form of various geopolymers found in asphalt waste. Respondents included government, design and research centres, asphalt mix manufacturers, and others directly involved in the production process. The number of respondents was more than 300. Given the narrow specialisation and subject matter of the survey, this number should be considered satisfactory. The responses received came from all over Poland. The largest number of questionnaires was received from the Świętokrzyskie Voivodeship, where the number of innovations and patents on the use of waste is very high. The number of respondents by province is shown in Figure 1.

The purpose of the survey was to determine the extent of the needs and problems faced by participants in the construction process who use a destructive material containing milled geogrid (geopolymer). Accordingly, five questions were selected from the entire survey to answer the research questions. Their list is presented below:

- Is it necessary to use RAP for asphalt mixtures?
- Is the geogrid in the RAP a technological problem?
- Has a problem been observed with the milling of the geogrid RAP?
- Does the geogrid contained in the RAP hinder technical acceptance?
- Indicate the observed grid size in the RAP and how it was removed.

**Figure 2:**
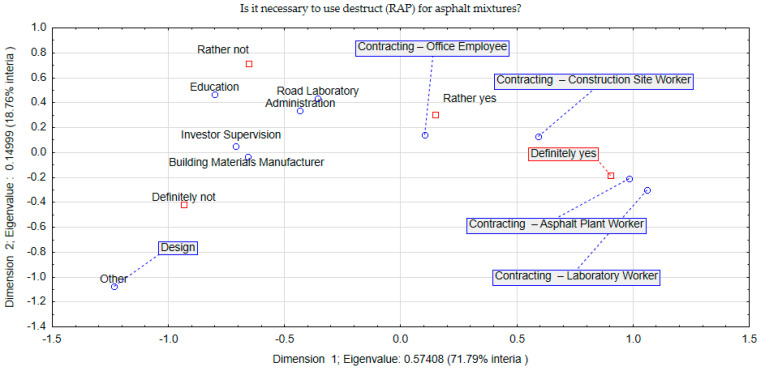
Correspondence (space) analysis concerning question 1.

The variables in the survey are qualitative and in order to quantify them, it was necessary to determine the number of occurrences of a given response as a percentage. For this purpose, the Burt table was used [33]. Within the scope of questions, which sought to obtain knowledge about the degree of acceptance of phenomena, views, processes, features, a Likert scale was used [34]. In order to correctly determine the scale’s ability to differentiate respondents’ attitudes, five levels were established for it: definitely no, rather no, do not know, rather yes, definitely yes. In some cases, the answer: do not know was dropped, as its result would distort the conclusions sought or cast doubt on the competence of the respondent to deal with pavement recycling issues. In addition to the frequency of responses given by each group of respondents, it was also important to look for relationships between groups of respondents and their responses. Such a complex qualitative analysis was performed using correspondence analysis, drawing on Hill’s experience [33,35]. Correspondence analysis is a descriptive exploratory technique that provides information about the structure of relationships between rows and columns from a multivariate table. It is the need to look for relationships between responses and groups of respondents in a generalised way that will be used to interpret survey results. By projecting the survey results in relation to groups of respondents, it is possible, by assessing their neighbourhood, to identify latent relationships between them. As a result, in terms of question 1, the distribution of respondents’ answers in terms of correspondence (space) analysis is represented by the graph below (Figure 2).

**Figure 3:**
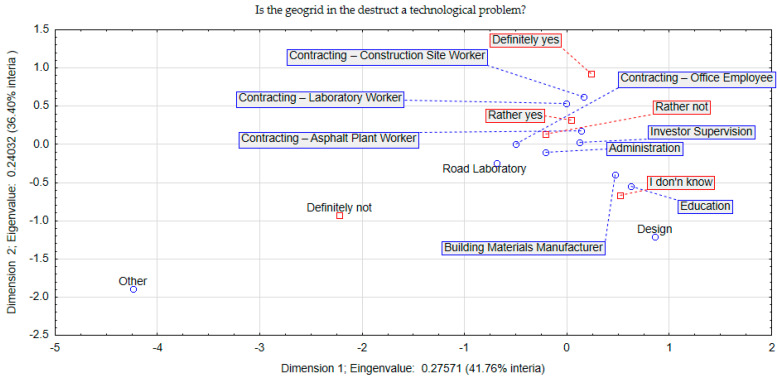
Correspondence (space) analysis concerning question 2.

The analysis was limited to two dimensions, which explained almost 91% of the variability in the survey data set. According to Figure 2, a strong endorsement of the use of destructive material for mineral–asphalt mixtures is shown by respondents involved in the manufacture and incorporation of mineral–asphalt mixtures. These include the technologists who produce the mineral–asphalt mixture and the engineers who supervise the incorporation of the mineral–asphalt mixture on the road section. Thus, these are the people who are directly involved in solving technical and economic problems on the construction site. Dimension 1 divides respondents into groups that are keen to use destructive material (contractors) and groups that strongly deny the need for its implementation. In the second group, with a reluctant attitude, are respondents representing construction supervision and designers. In the case of construction supervision, the use of destructive material always entails the risk of disrupting the uniformity of its use and the uncertainty of maintaining the designed durability of the mineral–asphalt mixture. In the case of designers, the location of this group in Figure 2 (coordinates) and the aforementioned reluctance are not due to problems related to, e.g., acceptance or legislative elements, but probably to a lower awareness and experience in its application. Therefore, conducting further research related to the implementation of a destructive material containing geogrid into the mineral–asphalt mixture for its wider use is justified. The groups with the most indeterminate opinion are education and administration. In this case, this is due to the specificity of the activities carried out, which require the application of procedural and legal conditions. Nevertheless, the aspect of lower awareness also emerges, as in the case of the design group. The obtained conclusions were complemented by the information resulting from the answers given to question No. 2, which is presented in Figure 3.

The space indicated explained 79% of the relationships within question 2. According to Figure 3, the group of workers directly involved in the execution work and the laboratory units indicated a definite problem with the presence of geogrid contamination in the RAP. It should be borne in mind that the current popularity of the use of geogrid to reinforce road structures is a very common phenomenon. So, the problem reported by contractors manifesting itself in the occurrence of RAP with geogrid certainly cannot be ignored. In the case of the design group, their response was unspecified (I do not know). On the other hand, observing the distance of the centroid of the answers of the designers’ group in relation to the coordinates of the centroids of the answers of the other groups, it can be concluded that their position was definitely due to less experience in the removal of geogrid from the destructive material.

**Figure 4:**
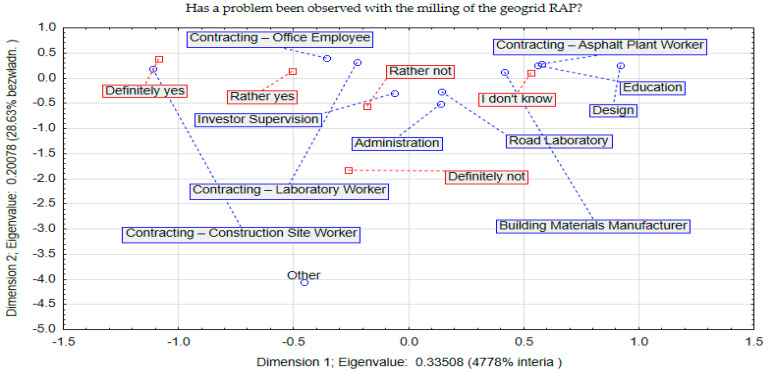
Correspondence (space) analysis concerning question 3.

Since the respondents report technological problems with the presence of geogrid contamination, it is important to assess whether there are technical problems when milling asphalt pavements containing geogrid. This range of information was contained in the answers to question 3. The correspondence analysis is shown graphically in Figure 4.

Analysing the results shown in Figure 4 regarding the technical problem during milling of the geogrid-reinforced destructive, it can be seen that it was reported by contractors on site. This is an obvious result, as the problem of cleaning the milling drums is the main problem when milling mineral–asphalt mixture reinforced with geogrid. The constructed space of the correspondence analysis explained the relationships occurring in the set at a level of 77%. In the case of investor supervision, the technical problem was not indicated as significant. This is probably due to the fact that the technical solutions related to the removal of the geogrid and the maintenance of the schedule of the construction works lie most with the contractors. Therefore, this type of problem is not directly within the competence of the construction supervision. Similar technical problems are not perceived by the education-related centres and the design group. For the “I do not know” response, there was also a large group of respondents related to the employees of asphalt plants and construction product manufacturers, whose job is not to mill a mineral–asphalt mixture.

**Figure 5:**
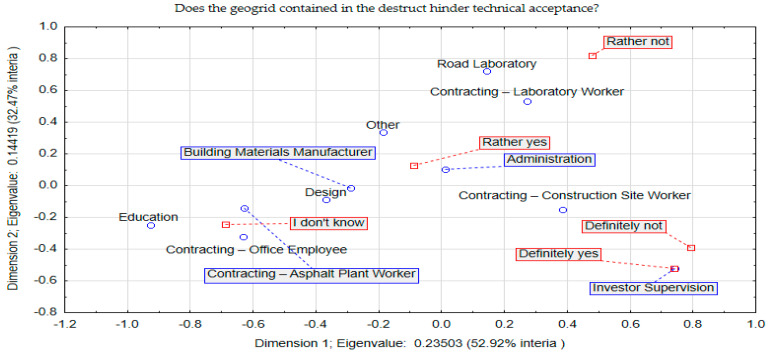
Correspondence (space) analysis concerning question 4.

A question that also fell within the scope of the questionnaire concerned reported problems during the acceptance of a layer with a destructive material containing a geogrid. This question was assigned No. 4, while the corresponding results of the correspondence analysis are shown in Figure 5.

A definite problem with the acceptance of mineral–asphalt mixture containing a visible amount of geogrid was reported by the investor supervision (answer “Definitely yes”). This was an expected result, as the role of supervision is to take care of the quality of the completed pavement, while applying the technical and legal provisions. In the absence of comprehensive information and certainty about the implementation of a significant amount of geogrid and its type, the technical supervision usually issues a negative decision regarding the acceptance of the pavement layer in question or requires an additional expert opinion. The appearance of some contradiction in the form of a “definitely not” answer should be regarded as the noise of the research process, in which respondents with a certain profile probably accidentally marked an answer that did not correspond to their line of transverse answers. In contrast, road lab staff did not observe problems with collection as they are not involved in the collection process. A legislative problem is also reported by a group of administrative staff. In the absence of additional provisions in the technical specifications, the excess dirt (according to WT-2/2014 [22]) is clearly interpreted (negatively) during technical acceptance.

**Figure 6:**
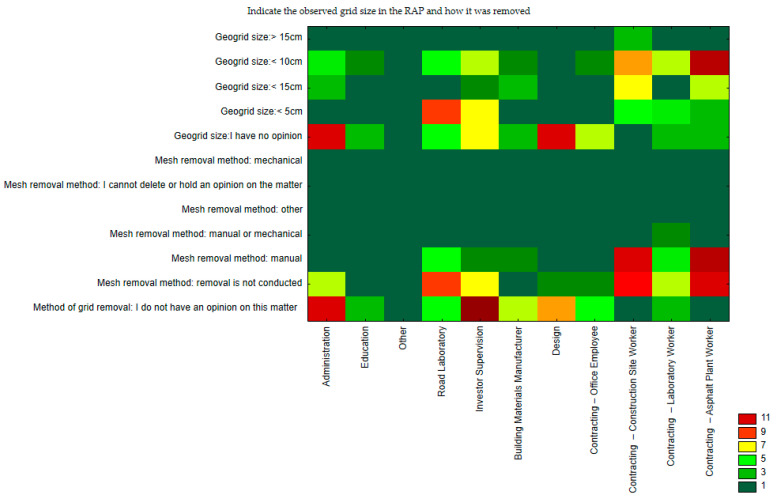
Correspondence (space) analysis concerning question 5.

The next and final question (No. 5) concerned the opinions of the respondent groups on the extent of geogrid contamination and the method of disposal. In this case, the analysis of the respondents’ answers was performed using a sequential graph. The percentage distribution of respondents’ answers is shown in Figure 6.

From the sequence diagram in Figure 6, it can be seen that the workers at the plant remove debris from the geonet by hand. However, they mostly deal with grids up to 10 cm. For the road laboratory staff as well as for the project supervision, objects up to 5 cm in size of geonet contamination are frequently observed. In this respect, the administration, the designers, and above all the investor supervision have not made a clear statement. Thus, the problem of contamination removal remains the responsibility of the participants involved in the manufacture of mineral–asphalt mixture. It is also apparent from the graph in Figure 6 that a certain small proportion of respondents employed directly on the construction site (contracting) sometimes have to deal with a geogrid size larger than 15 cm. In summary, the answers given to question 5 indicate that the most common geonet size is an object in the range of 5 to 10 cm. In contrast, its removal is done manually, or no additional action is taken. In conclusion, the responses to question number 5 reveal that the predominant size of a geogrid is within the range of 5 to 10 cm. Nevertheless, its removal is conducted manually, with no further measures employed.

The information that emerges from the questionnaire, and in particular from question 5, suggests the need for a study that will answer the following question: what consequences may arise from leaving some geogrid objects of milling mineral–asphalt mixture in excess of 0.1%. Thus, the results obtained will allow a broader view of the consequences for the quality of the mineral–asphalt mixture mineral–asphaltdue to the presence of geogrid contamination in RAP and will allow preliminary recommendations to be made.

## 3. Materials and Methods

### 3.1. Geogrid

**Figure 7:**
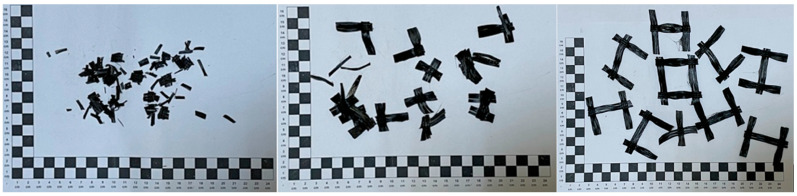
Micro and macro glass fibres (one square equals one centimeter).

**Figure 8:**
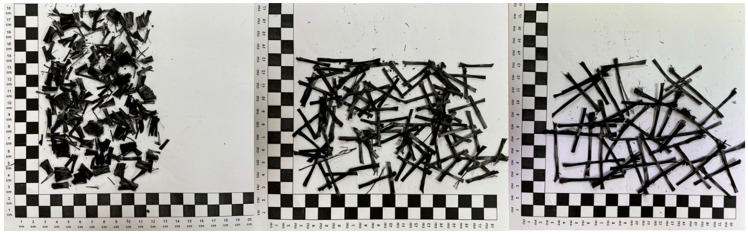
Micro and macro glass fibres (one square equals one centimeter).

The selection of mesh type for this study was informed by survey results and insights gained from our RAP analysis. Glass and carbon fibres are the predominant materials used in geogrid construction. Consequently, both types of fibres were incorporated into the research to accurately represent the existing conditions. The determination of the type of fibre in relation to its size depends on the largest dimension of the aggregate D in the mineral and asphalt mixture. In relation to the grain size of AC16W and AC22W mixtures, micro fibres are those fibres whose length is less than 16 mm or 22 mm depending on the type of mixture. Macro fibres are those whose size is greater than 16 mm or 22 mm depending on the mix type. As part of this study, micro and macro fibres from glass and carbon geonets were introduced into the asphalt mix. The geonets from which the fibres were extracted were designed for use in asphalt pavements and overlays and meet the Polish requirements of EN 15381 [36]. The fibres were cut to a length of 1 cm—micro size, and 3 cm and 5 cm—macro size. Figure 7 shows the glass fibres and Figure 8 the carbon fibres.

In addition, as a supplement, Table 1 shows the declared properties of the glass and carbon geogrid used.

**Figure 9:**
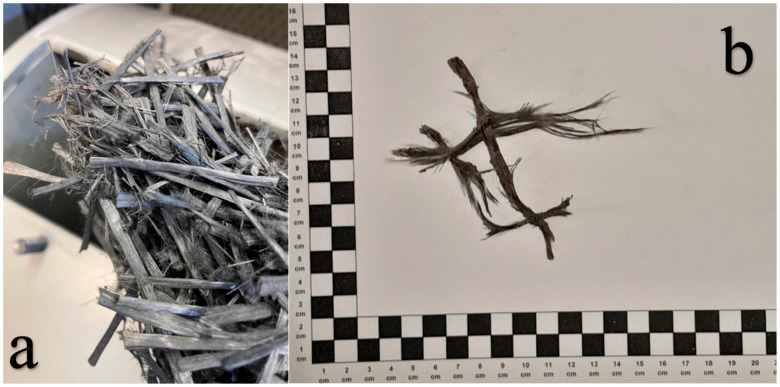
Geogrid fibres after the ageing process and pavement operation (one square equals one centimeter). (**a**) new geogrid fibres after the ageing process in the Los Angeles drum; (**b**) single geogrid fibre after asphalt pavement milling process.

The properties of the geogrids given are taken from the manufacturers’ declarations of the geosynthetics supplied and are the minimum, maximum, or range of results. For the purposes of this research, the treatment of the fibres included, in addition to cutting them to size, performing a process of ageing them in a Los Angeles drum (Figure 9a). The mechanism for ageing the fibres consisted of a cycle of 1500 revolutions of the Los Angeles drum. During one cycle, 1 kg portions of new geogrid fibres were aged. Examples of actual fibres after mechanical removal of asphalt layers with a reinforcing layer (geogrid) are shown in Figure 9b. In contrast, the fibres after the ageing process are shown in Figure 9a.

The ageing and trimming to appropriate dimensions of the new geogrid fibres under laboratory conditions is a procedure that is intended to replicate the many years of service of the geogrid in the road pavement and the geometric form that is most often found in the asphalt RAP after the geogrid milling process. From the point of view of the planned experiment, the effect of geogrid wear will definitely improve the quality of the prediction of the laboratory results.

### 3.2. Asphalt Concrete Design

**Figure 10:**
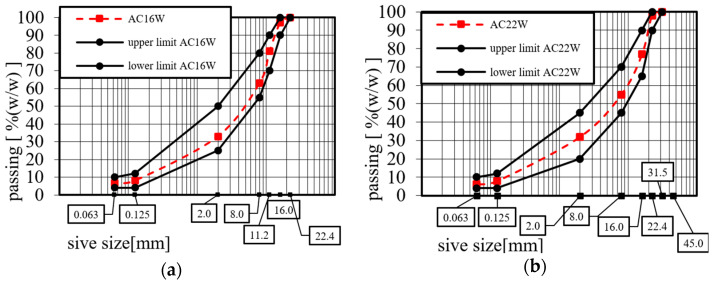
Grain size curve of asphalt concrete (AC): (**a**) AC 16W KR3-4; (**b**) Grain size limit curves of AC 22W KR3-4.

The design of the asphalt concrete (AC) recipes was preceded by a detailed analysis of all the mineral components, asphalt granules, the asphalt binder used, and the additive of glass and carbon fibre geogrids in terms of compliance with the requirements of standards for materials intended for AC produced using hot-mix technology. The designed reference mineral–asphalt mixtures, which were used for the study, have been in operation for many years in the road network space and are widely used for the construction of local roads in the świętokrzyskie voivodship and neighbouring voivodships. The quality of the asphalt layers made, using the asphalt concrete prescriptions referred to, for the binder course of a road designed for KR3-4 traffic (ESAL_100kN_ from 0.5 to 7.3 million) [37], has been repeatedly confirmed in acceptance tests following completion of the contract works. The method of limit curves was used to design the composition of the mineral mixture. The grain size curves of the mineral and asphalt mixtures used in the study AC 16W KR3-4 and AC 22W KR3-4 are shown in Figure 10a,b.

**Figure 11:**
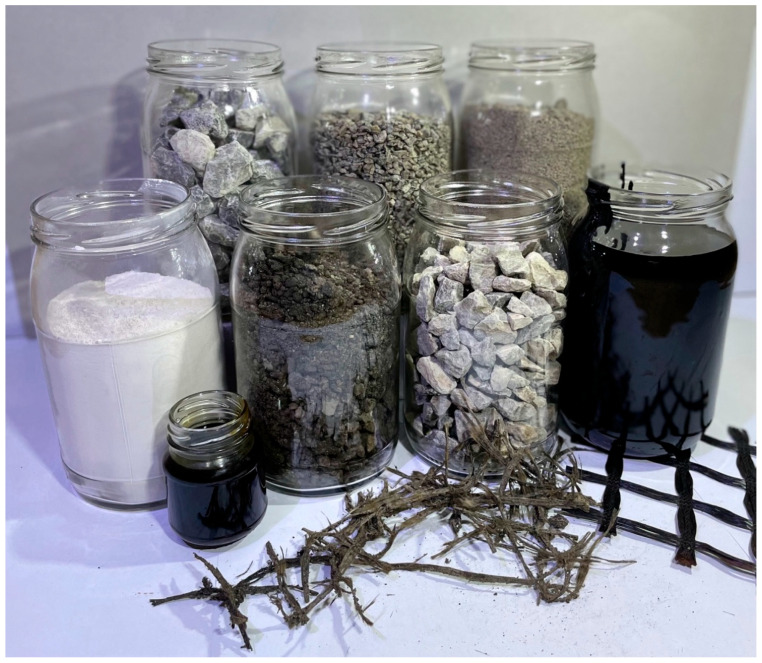
Materials used in this study.

The selection of mixtures with grain sizes of 0/16 and 0/22 was determined by the guidelines outlined in the Polish catalogue of typical asphalt pavements [37] and WT-2/2014, which are applicable for vehicular traffic loads specified from 0.5 to 7.3 million ESAL100kN. Moreover, the selection of mineral and asphalt mixtures for the binding course was influenced by the frequent incorporation of RAP into this composite type. Based on the limit curves developed in Technical Guideline WT-2/2014 and research in work [38], the AC 16W reference mineral mix was assumed to contain 20% granulated reclaimed asphalt pavement (11.2 GRA 0/8.0), 37% 8/16 mm coarse aggregate, 21% 2/8 mm coarse aggregate, 21% 0/2 mm fine aggregate, and 1.0% limestone filler. The designed reference AC 22W asphalt concrete mix contained 20% granulated reclaimed asphalt pavement (22.4 GRA 0/16), 22% 16/22 mm coarse aggregate, 20% 8/16 mm coarse aggregate, 16% 2/8 mm coarse aggregate, 21% 0/2 mm fine aggregate, and 1.0% limestone filler. Materials used were sourced from Świętokrzyskie dolomite and limestone aggregate plants. The materials used during testing are shown in Figure 11.

The single batch was 10 kg, and the materials were mixed mechanically at predetermined ratios, respectively, for AC 16W and AC 22W according to the procedure described in standard EN 12697-35 [39]. As the proportions referred to reference mixes without the addition of fibres, each time a mix was tested with the corresponding code symbol, the appropriate type of geogrid fibre (glass, carbon), the appropriate fibre percentage for the replication (0.2, 0.6, 1.0%), and the assigned fibre length (1, 3, 5 cm) were implemented into the mixer, as determined by the established experimental plan. For the asphalt mixes, 35/50 road asphalt was used at 3.7% for AC 16W and 3.4% for AC 22W. The remaining required amount of total asphalt was supplemented by the asphalt contained in the asphalt granules and was 0.9% for the AC 16W mix and 1.0% for the AC 22W mix. It should be noted that the volume dependence resulting from the addition of geogrid recycled from glass and carbon fibres was not taken into account in the design of the asphalt concrete mixes. This procedure was intended to simulate, as far as possible, the situation in which the geogrid fibres together with the waste material enter the AC composition at the asphalt mixing plants in an uncontrolled manner. An adhesion agent (fatty acid amides) was added in the design of the mineral–asphalt mixture to ensure adequate adhesion of the dolomitic aggregate to the 35/50 road asphalt and the asphalt derived from the asphalt granules. Its main function is to prevent the asphalt from separating from the aggregate, especially under conditions of increased moisture. By changing the chemical–physical nature of the contact, the surface tension of the asphalt between the aggregate and asphalt is reduced, improving the surrounding of the aggregate with the asphalt. This is key to ensuring the durability of road surfaces, making them more resistant to water. An asphalt waste material from the wearing and binder course of a provincial road was also used for laboratory testing of the mineral and asphalt mixtures due to its nature as a carrier of geogrid dirt. From the AC batches thus made in the mixer, samples were formed for the individual types of test, in accordance with the requirements specified in the respective test method.

### 3.3. Experimental Design and Modelling of Asphalt Concrete Properties Using a Generalised Linear Model (GLM)

A classical factorial experimental design was used to assess the effect of the geogrid on the properties of asphalt concrete. The sampling scheme adopted was important for the prediction of the dependent variables and influenced the formulation of conclusions. The starting topology of the experiment plan was an experiment plan with different values of bivariate and trivariate input quantities. These experimental designs are an amalgamation of the procedures from fractional factorial plans of types 2^(k-p)^ and 3^(k-p)^, as classified by Connor and Young [40]. The percentage range of the geogrid incorporation was determined in accordance with the provisions of the applicable WT-2/2014 regulations. On the other hand, the fibre lengths were derived from survey data, which indicated a typical length of approximately 5 cm. The initial parameters for the mixing process constituted the experimental domain, encompassing the independent (controlled) variables detailed in Table 2.

The two quantitative variables were entered at 3 levels and the other two qualitative variables at 2 levels. For the qualitative variables, additional coding was required. This procedure was necessary to build the regression model, determined by the generalised linear model (GLM) algorithm widely described [41]. Accordingly, the AT mix type AC 16 W was assigned the value 0, while AC 22 W was coded as 1. In the same way, the levels of the GT variable were coded: carbon mesh 0, and glass mesh 1. The choice of model was dictated primarily by the number of effects that were taken into account. It was assumed that there could be no non-linear effects of qualitative as well as quantitative variables on the dependent characteristics. It should be noted that the model adopted mainly took into account the possibility of assessing interactions between qualitative and quantitative variables. The regression model finally adopted had the general formula shown below (1):

(1) $$y=b_{0}+b_{1}x_{1}+\ldots b_{k}x_{k}+b_{12}x_{1}x_{2}+\ldots +b_{k-1.k}x_{k-1}x_{k}+\ldots +b_{11}x_{1}^{2}+\ldots +b_{kk}x_{k}^{2}$$

where *b_k_*—experimental coefficients; *x_k_*—variables; *y*—dependent variable. Taking into account the aforementioned limitations, the modified model (1) was further evaluated using D-optimal algorithms. Their task was to maximise the determinant of the information matrix XX [42]. This was a key measure to minimise the volume of the total confidence set of all regression coefficients and increase the efficiency of inference on the model parameters. The assessment of G-optimality [43] was evaluated using Equation (2):

(2) G-optimality=100×square root(p/N)1/2/SM

where p—the number of factor effects in the plan, while N is the number of required layouts; Σ_M_ denotes the maximum standard deviation in the predicted value of the dependent variable including all proposed points. The G-optimality criterion is defined as the plan that minimises the largest value of the standard deviation in the determinable response surface (of the dependent variable determined from the approximating function). As a result, an experimental plan containing 14 combinations was constructed to meet the conditions for maximising values according to the G-optimality criterion. It is worth mentioning that, if the full experimental plan had been used, the number of combinations (number of prescriptions) would have been 36. Each dependent feature was replicated between 2 and 6 times. The number of replications resulted from the standard requirements according to which this study was implemented. It was very important to randomise the order in which each combination was performed in order to minimise the bias attributed to the operator’s precision in performing successive mixtures. Therefore, the execution order of the mixtures was realised in a coded form after prior randomisation. Finally, the experimental domain used in this study is shown in Table 3.

The estimated efficiency value of the G-optimal characteristic was 71%. This value signifies the degree to which the modified experimental design captures the anticipated variability in the instance where a full factorial design would be applied. To elucidate the relationship between the characteristics of plastomer-modified asphalt and the process variables associated with mixing, the aforementioned generalised linear model (GLM) algorithm was utilised. Its syntax allows you to take into account variables of a quantitative nature, but also of a qualitative nature. The aforementioned GLM algorithm was used to model the relationship between the characteristics of the plastomer-modified asphalt and the mixing process variables. Another notable advantage of using GLMs is the inclusion of interaction effects and non-linear members in the function. Accordingly, model (1) was adopted as the test object function. In the present analysis, its form is derived using the binding function as an identity function, which has the task of appropriately relating the variables in the vector of values of the independent variables to the vector of expected values. In essence, the binding function is a form of transformation of the expected variable. In the case of the analysis performed, the adoption of the binding function was due to the fact that the distribution of the dependent characteristic was a normal distribution [41]. The generalised form of the adopted model had the following Formula (3):

(3) $$g\left(Y\right)=X\cdot \beta =\sum_{j=1}^{k}\beta_{j}x_{ji}+$$

where *Y*—vector of dependent variable values; *X*—matrix of independent variable values; ε—random component of residuals; *β*—vector of model coefficients; *g*—binding function. The final consideration was how to implement the qualitative variables. As a result, the qualitative variables were coded on a binary scale of 0–1 and were implemented in the regression model using the so-called ‘reparameterised model’. In this situation, a generalised inverse of the matrix X’X is required to determine the vector of model coefficients. The generalised inverse in the case of an incomplete-order matrix, due to the modification of the initial plan, was calculated by simply intentionally zeroing the elements in the redundant rows and columns of the matrix [44].

A notable characteristic of the generalised model, as previously mentioned, is the capability to incorporate categorical variables at two distinct levels (0 or 1), synonymous with a dichotomous variable. The procedure for determining the adjusted GLZ model parallels that of classical linear models. Initially, it necessitates the identification of an a priori model of the objective function. For this analysis, this corresponds to model (1). A critical component of the analysis involves determining the specificity of the binding function g(E(Y_i_)) as expressed in Formula (3). The identity function was adopted under the assumption that the distribution of the dependent variable E(Y_i_) follows a normal distribution. This assumption was informed by prior studies and the evaluation of the dependent variable’s distribution using the Shapiro–Wilk normality tests. It is noteworthy that a binding function serves as a mechanism for transforming the variable Y to stabilize its variance to the greatest extent possible. A pivotal aspect that differentiates the GLZ algorithm from the classical method is the approach for identifying the b_ij_ parameters within model (1). The GLZ model employs the Maximum Likelihood Estimation (MLE) method, which seeks the parameter estimates that optimize the likelihood of the observed data. In the context of the GLZ model, parameter estimation is conducted based on an n-element sample for which the values of y_1_, y_2_,…y_n_ were recorded. The credibility function assumes form (4).

(4) $$L\left(y_{i},\beta_{i},\varphi \right)=L\left(y,\beta \right)=\overset{n}{\underset{i=1}{\prod }}P\left(y_{i}|\beta_{i},\varphi \right)$$

where $P\left(y_{i}|\beta_{i},\varphi \right)$—the likelihood of the variable’s value occurrence within a specified model delineated by certain parameters$\;\beta_{i}$. This likelihood is contingent upon the chosen binding function. The process entails maximising the logarithm of the maximum likelihood as depicted in form (5):

(5) $$\frac{\partial }{\partial \left(y,\beta \right)}lnL\left(y_{i},\beta_{i},\varphi \right)=0$$

The implementation necessitates employing iterative numerical methodologies, specifically the Newton–Raphson method. Estimator determination presents significant challenges from an accounting perspective. Consequently, the utilisation of the libraries and predefined functions available within the Statistica package was deemed essential. Furthermore, the significance of the parameters in the derived model was assessed using Wald’s tests, as delineated by Formula (6):

(6) $$W={\left(\frac{\beta_{j}}{\sigma_{\beta_{j}}}\right)}^{2}$$

where $\beta_{j}$—the estimator of the j-th variable and $\sigma_{\beta_{j}}$—asymptotic standard error are concerned, the verification statistic follows a Chi-square distribution with one degree of freedom. Prior to evaluating the pertinent variables, an additional phase in the model construction process was implemented. This phase was intended to identify the optimal set of parameters, during which an additional Akaike criterion was applied to “penalize” the likelihood function, thereby favoring a more parsimonious model [45,46]. Two attributes were utilised to evaluate the model’s conformity to the experimental data: an additional qualitative metric for assessing the regression equation was the Root Mean Square Error (RMSE) and the coefficient of determination R^2^ [47,48]. The estimation of the R^2^ coefficient is articulated by Formula (7).

(7) $$R^{2}=1-\frac{\sum_{i=1}^{n}{\left(y_{i}-\hat{y_{i}}\right)}^{2}}{\sum_{i=1}^{n}{\left(y_{i}-\hat{y}\right)}^{2}}$$

where $y_{i}$—the experimental value pertaining to the i-th measurement of the dependent variable, $\hat{y}$—the arithmetic mean of the dependent variable, $\hat{y_{i}}$—the forecasted value that corresponds to the i-th measurement of the dependent variable. The RMSE signature is delineated by Formula (8):

(8) $$RMSE=\sqrt{\frac{\sum_{i=1}^{N}{\left(y_{i}-y_{mean}\right)}^{2}}{N-1}}$$

where *y_i_*—ith observed value, *y_mean_*—mean value, *N*—number of trials.

### 3.4. Research Method

Four characteristics were included as part of this study to compare the resulting mixtures with mesh-contaminated reclaimed asphalt pavement (RAP) to the reference asphalt concrete mixtures within the requirements of WT-2/2014. Therefore, the following were selected as the critical set of characteristics to be determined:

- Free space (V_a_),
- Stiffness modulus (Sm) at 13 °C,
- Water resistance (ITSR),
- Rutting resistance (WTS_AIR_, PRD _AIR_).

#### 3.4.1. Void Content

The void content (V_a_) plays an important role in the mineral–asphalt mixture. Too high an amount results in a decrease in water and frost resistance, while a low amount can contribute to a reduction in resistance to permanent deformation and in indirect tensile strength. This parameter is determined according to EN 12697-8 [48] as the volume of air voids in the specimen, which is expressed as a percentage of the total volume of the specimen according to Formula (9):

(9) $$V_{a}=\frac{\rho_{m}-\rho_{b}}{\rho_{m}}\cdot 100\%$$

where

$V_{a}$—free space content (0.1%),

$\rho_{m}$—density of the mineral–asphalt mixture (Mg/m^3^),

$\rho_{b}$—volumetric density of the mineral–asphalt mixture (Mg/m^3^).

The density of the asphalt mixture determined according to EN 12697-5:2010 [49] was used to determine the void content using pycnometric procedure A ($\rho_{mv}$), while the volumetric density was determined according to EN 12697-6:2012 [50] procedure A ($\rho_{bdry}$). The preparation of the specimens for the test consisted of compacting them under laboratory conditions with a Marshall rammer using 75 blows per side (2 × 75) according to EN 12697-30 [51].

#### 3.4.2. Stiffness Modulus

The determination of the stiffness modulus in the IT-CY intermediate tensile scheme (S_m_) was carried out for specimens conditioned under air-dry conditions at 28 and then at 56 days after compaction. The test was performed at +13 °C (equivalent temperature in Poland), for both conditioning periods according to EN 12697-26 [52]. The test temperatures corresponded to the average temperature of the winter period and successively to the temperature of the winter/spring period, the beginning of spring, the end of spring, and the summer period. The samples were compacted with a Marshall rammer using 75 blows per side (2 × 75) [51]. Immediately prior to testing, the samples were conditioned for a minimum of 4 h at the test temperature, then two sample measurements were taken along diameters intersecting at right angles.

#### 3.4.3. Water Resistance

Water and frost resistance testing was performed according to EN 12697-12:2008 [53] and according to Annex 1 of WT-2:2014 [54]. For the determination of the ITSR index, the samples were subjected to conditioning in water and 1 freeze–thaw cycle. The index was determined on the samples 28 days after compaction. Strength tests were performed at 25 °C (±1 °C), and the result was calculated according to Formula (10):

(10) $$ITSR=\frac{ITSR_{WET}}{ITS_{DRY}}$$

where

ITSR—Water and frost resistance [%],

$ITSR_{WET}$—indirect tensile strength at 25 °C of conditioned specimens (kPa),

$ITS_{DRY}$-indirect tensile strength at 25 °C of unconditioned specimens (kPa).

#### 3.4.4. Resistance to Rutting

Tests for resistance to permanent deformation were performed in a small rutting gauge according to EN 12697-22 [55]. The method consists of subjecting a single layer of the compacted mineral and asphalt mixture sample to repeated passes by a standardised pneumatic wheel until 10,000 passes are reached, or a rut depth of 20 mm is reached in the sample at a temperature of 60 °C. At the end of the test, the required parameters are calculated in accordance with EN 12697-22 [55]:

- proportional rut depth PRD_AIR_,
- rut growth rate WTS_AIR._

The test was carried out on two slabs of each type of mineral and asphalt mix.

## 4. Results

### 4.1. Evaluation of the Influence of Controlled Factors on the Properties of Asphalt Concrete

**Figure 12:**
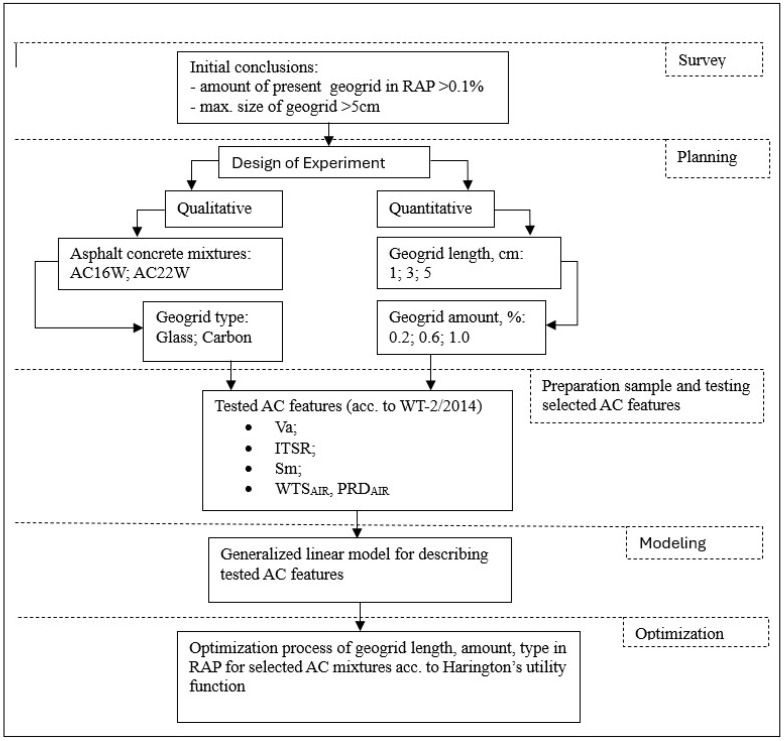
Flowchart of experiment setup.

The researchers’ methodology encompassed multiple stages, spanning from the articulation of the problem statement to the formulation of recommendations for the final material, in alignment with established best practices [56]. The structure is depicted in a flowchart, as illustrated in Figure 12.

Figure 12 illustrates the sets of predictors and dependent variables that have been identified based on the employed topology of the Design of Experiments (DoEs) composite factor plan. The mathematical explication of the results acquired during the research played a pivotal role. The relevant parameters of the GLM (3) were determined using the method of identifying the best subset of effects. It consisted of performing a search for all possible subsets of effects occurring within the current system (individual as well as interaction effects). The method of searching for the best subsets was based on the Akaike information criterion [57]. When fitting a model with q parameters to the data, this criterion is defined as −2Lq + 2q, where Lq is the maximum of the logarithm of the confidence quotient. Akaike proposed that for models with different numbers of parameters, the one for which the expression value is maximum should be chosen. The Wald statistic was used to eliminate irrelevant parameters from the model [58]. Initially, the criterion was used for time series models, but it is also used in regression. This yielded sets of parameters assigned to specific regression models describing all the characteristics considered in this study. The final formulation of the regression models was made on the basis of the realised experimental plan given in Table 3. The quality of fit and the mathematical notation of the regression models are presented together with the accuracy of the measurements (RMSE) and the evaluation of the convergence of the results (R^2^) in Table 4.

In order to make a prediction about the value of a feature, it is useful to use an example. The WTS_AIR_ was chosen as an example. The notation of the regression model (GLZ) for the WTS_AIR_ based on Table 4 is of form (11):

(11) $$WTS_{AIR}=0.039696+0.105586890\cdot GP-0.024184451\cdot AT\cdot GP+0.008934451\cdot AT$$

Taking into account the previously mentioned and assigned code values for quality characteristics:

- MMA(AT) Mixture Type: 0—AC16W, 1—AC22W;
- Type of Geogrid (GT): 0—carbon mesh, 1—glass mesh.

Equation (11) for the WTS_AIR_ for the AC22W mix and carbon mesh ultimately has (unsimplified) Formula (12):

(12) $$WTS_{AIR}=0.039696+0.105586890\cdot GP-0.024184451\cdot 1\cdot GP+0.008934451\cdot 1-0.004475610\cdot 0\cdot GL$$

**Figure 13:**
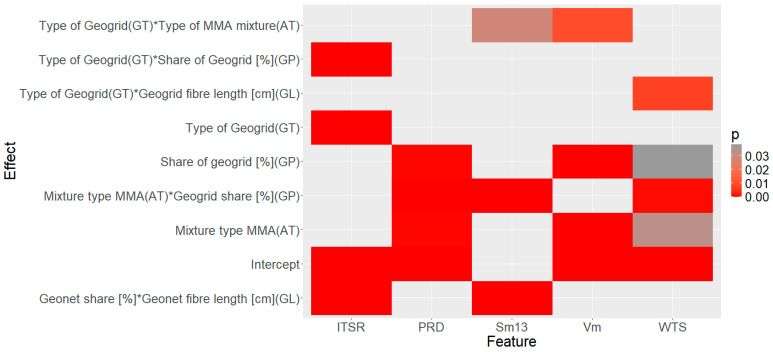
Gradient plot of the *p*-value of the tested asphalt concrete characteristics.

In the example presented, no rounding or simplification was applied. The number of decimal places resulted from the assumed default settings. In this way, the generalised equation can be used to predict the behaviour of a material in relation to a given feature with varying structure, also taking into account qualitative cases. It should also be noted that the interpretation and assessment of the significance of the parameters (*p*-value) would be difficult if only the notation in Table 4 was used. Therefore, an additional assessment of significance, consistent with Table 4, is presented graphically in Figure 13.

It should be emphasised that the assessment of the significance of the parameters obtained from the GLZ model was juxtaposed with a series of successive plots of variability developed from the experimental data (Exper.). Such an exercise was intended to clarify the phenomenology of the results obtained in the laboratory and to contrast them with the effects identified using the GLZ technique in Figure 13.

**Figure 14:**
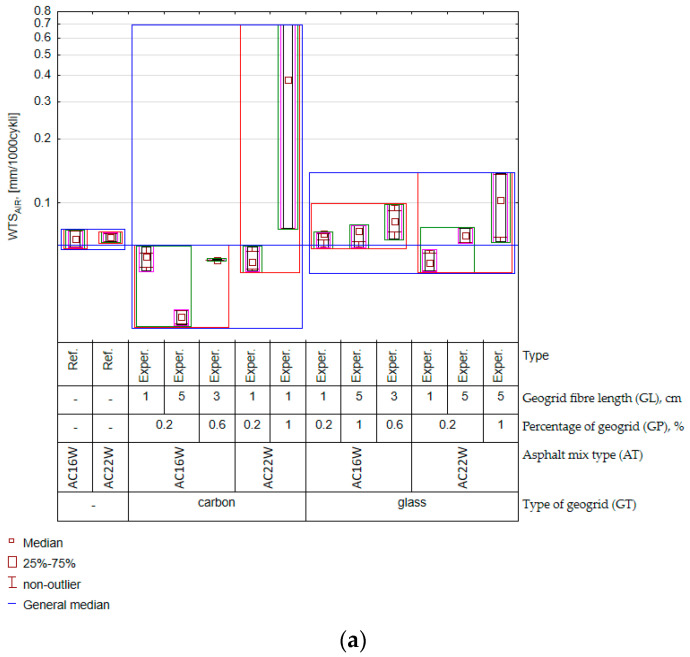

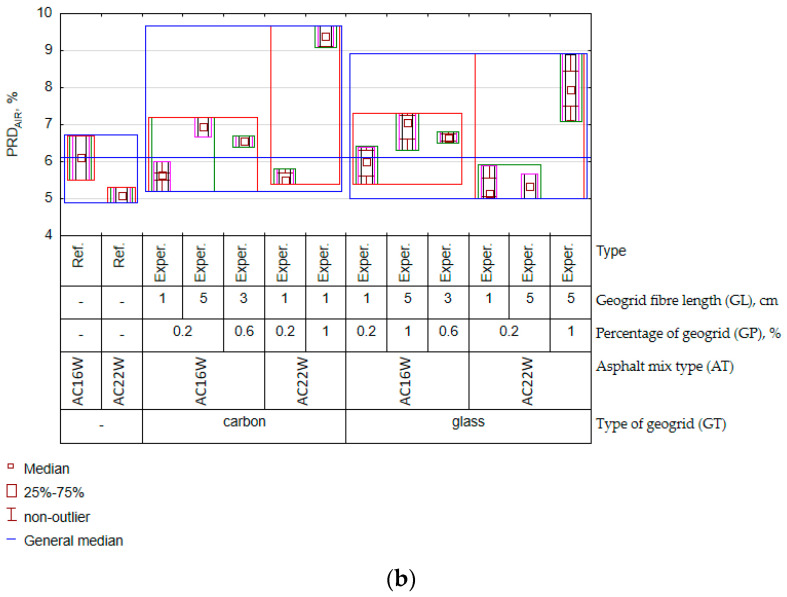
Feature variability charts (experimental results): (**a**) WTS_AIR_; (**b**) PRD_AIR._

In this connection, it should first be noted that the type of mix and the percentage of geogrid proved to be significant effects to be taken into account for the feature Sm and WTS_AIR_ and PRD_AIR_ (based on Figure 13). This conclusion, in the case of the features WTS_AIR_ and PRD_AIR_, is confirmed by the results aggregated in the variation graph of the experimental results (Figure 14).

**Figure 15:**
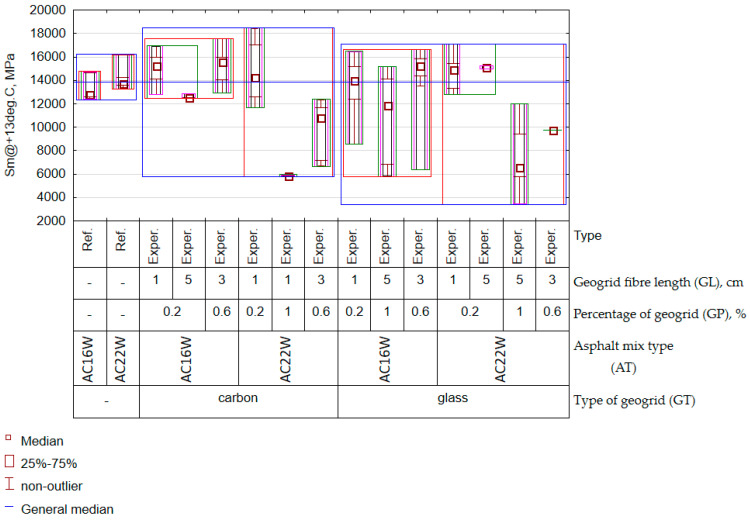
Graph of variation for the Sm at 13 °C (experimental results).

It should be added that the results of the rutting characteristics for the cases complying with the experimental plan were compared with the results obtained for the reference AC mixture (Ref.) In the case of the characteristics WTS_AIR_ (Figure 14a) and PRD_AIR_ (Figure 14b), observing Figure 13, there was an interaction effect between the length of the fibre and the type of geogrid. Thus, fibre length was important in the context of asphalt concrete creep, but the strength of the effect was determined by the type of fibre material. The reason for this may have been the relationship between the grain size of the mix and the number of fibres that were located between them. A similar relationship is observed in the selection of dispersed reinforcement in cement concrete. In this case, it appears that the length of the fibre added to the cement concrete should be approximately 1.5 to 3 times longer than the maximum aggregate grain size [59]. In the case of the AC16W mix, the indicated length of the added recycled mesh up to 5 cm based on the results of the characteristics WTS_AIR_ and PRD_AIR_ proved to be more appropriate in terms of the obtained spatial structure of the geogrid fibres than in the case of AC22W. It should also be noted that, compared to the reference mix (AC11W and AC22W), a reduction in creep rate (WTS_AIR_) was observed primarily for the carbon mesh and only in some cases for the glass mesh (AC22W, GP = 0.2%, GL = 1 cm). This shows that the presence of a certain amount of milled geogrid in the RAP does not necessarily reduce the quality of the material. The importance of fibre length, mentioned earlier, was also crucial for the assessment of the stiffness modulus (Sm). In the case of the Sm characteristic, this factor remained interactively entangled with the amount of mesh factor. The results of the distribution of experimental results were aggregated and are presented in Figure 15.

**Figure 16:**
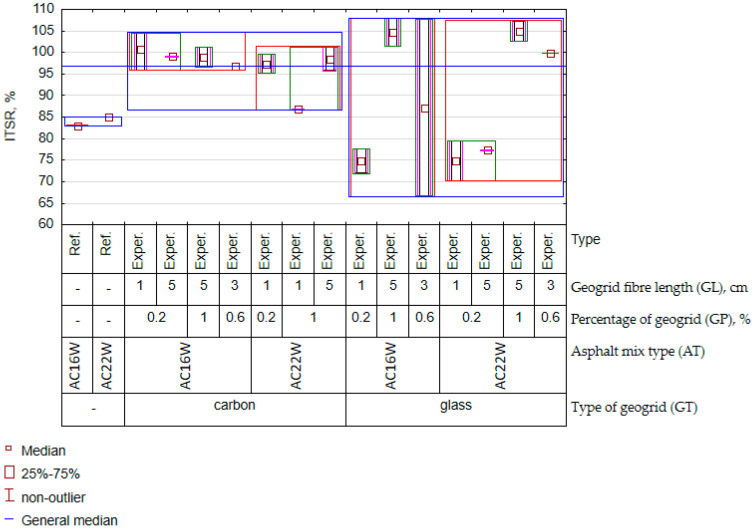
Graph of variation for the ITSR (experimental results).

In the case of the Sm, the change in the type of mineral mixture, in which the maximum dimension of the maximum grain changed from 16 mm to 22 m, resulted in a decrease in its values. In addition, the use of carbon fibre caused the Sm results to increase slightly. An interesting observation was that, during both the analysis of the WTS_AIR_, PRD_AIR_, and Sm, the mineral–asphalt mixtures containing glass fibre caused more variability in the results than the application of carbon fibre mesh. It was noteworthy that, in all cases, the application of carbon fibre mesh had a beneficial effect on the mechanical characteristics of the asphalt concretes. However, it should be remembered that its high quantity usually resulted in a deterioration of stiffness and resistance to permanent deformation, especially in the case of the AC22W mix. In contrast, an increase in fibre length for the finer AC16W mix proved to be a beneficial factor in reducing deformation rate (WTS_AIR_) and percentage rut depth (PRD_AIR_). Compared to the reference results, the presence of the carbon fibre geogrid did not significantly increase the Sm characteristic. In contrast, there was a decrease in stiffness for specimens containing glass fibre geogrid in many combinations. Considering the above observations, it should be noted that generalising the effect of geogrid fibres in asphalt waste to asphalt concrete without knowing the material of the geogrid is not the right approach. These observations will be taken into account in the search for an optimal solution for the number and length of geogrid fibres. In European conditions, not only mechanical characteristics are an important factor, but also the assessment of resistance to climatic factors. One of these is the water sensitivity of the ITSR. The distribution of the results in the form of a variation graph is shown in Figure 16.

**Figure 17:**
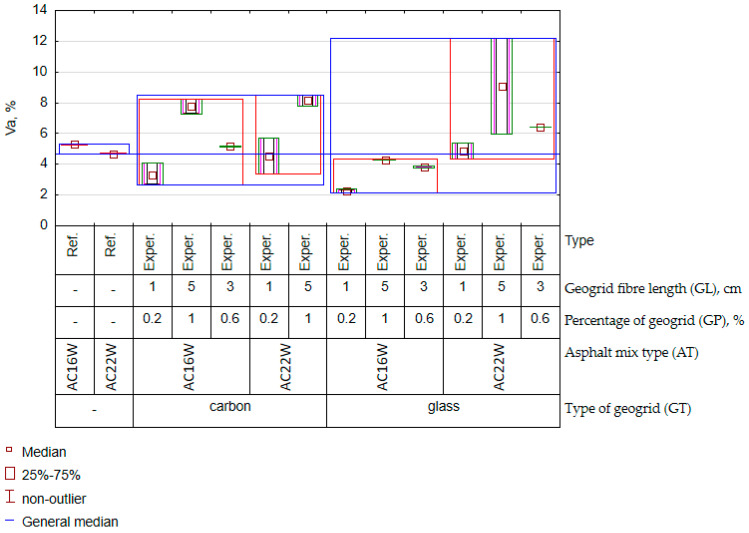
Variation graph of V_a_ (experimental results).

Observing the results of the variation of the ITSR (water resistance) in Figure 16 and the results of the statistical analysis contained in Figure 13, the most significant factor influencing its value was the type of geogrid. The next most significant factors remaining entangled were the length and the number of fibres. In the literature, it is possible to find information indicating that the material from which the fibres for mineral–asphalt mixture reinforcement are made have different absorption of asphalt [60]. Also, for the geogrids analysed, a significant influence of a factor was observed: the type of geogrid. In contrast, no significant influence of the type of mix was observed, i.e., no significant difference was observed between the ITSR results for the yields assigned to AC16W and AC22W. In this case, it was not the mineral skeleton but the geometrical properties and material of the geogrid that played a much greater role. Therefore, it can be assumed that the size of the geogrid elements, i.e., their length and number, influences the nature of the affinity with the asphalt and, consequently, the tightness of the mineral–asphalt mixture. This is particularly important, as the relationship between granulation and geometry of the recycled mesh was more significant for mechanical characteristics than for water sensitivity. Comparing the results of the ITSR parameter between the experimental results and the reference samples, the presence of the geogrid definitely increased the water resistance of the AC when carbon fibre was applied to the asphalt concrete. The same conclusion can be drawn for some cases of asphalt concrete where glass fibre was used for glass fibre (GP = 1%, GL = 5 cm). The reinforcing function of the geogrid, which increased the ductility of the mixtures during the indirect tensile strength test—the characteristic on which the ITSR result is calculated—is probably responsible for this fact. The water resistance is related to the porosity of the mineral–asphalt mixture. In this sense, the void content will also change when dosing different amounts of recycled geogrid. The experimental results of the change in void content are shown in Figure 17.

Only two effects played a significant role in the variability in the feature V_a_ (Figure 17), namely the type of AC mix and the type of geogrid. Also, part of the variability in V_a_ was explained by the interaction between the two (Figure 13). It should be noted that the void content (V_a_) increases as the number of fibres and their length increases. This trend is the same in both mixtures with the difference that the growth rate of V_a_ is higher when using glass geogrids than when using carbon fibre geogrids. This probably has to do with the increased absorption of asphalt by the glass mesh. As a result, the amount of asphalt absorbed by the geogrid resulted in a relative decrease in the volume of asphalt in relation to the volume of the aggregate and caused an increase in the voids in the asphalt concrete. This peculiarity is far more pronounced if the AC16W and AC22W mixtures where the glass mesh was used are juxtaposed. In the case of the carbon fibre geogrid, the effect of mix type was negligible. Based on the evaluation of the ITSR and V_a_ parameters, the effect of the type of geogrid can also be attributed to the milling process, where abrasion between the aggregate and the geogrid (also simulated in the laboratory) will induce a change in the surface area of the fibres and thus an increase in their specific surface area. In the case of the glass mesh, the erosion process was probably found to be more important than in the case of the carbon fibre mesh. However, in order to determine the range of geometrical parameters of geogrids (contamination) that can be considered acceptable in AC in conjunction with the type of polymer from which they are made, a multi-criteria optimisation process must be carried out.

### 4.2. Optimisation of the Quantity and Fibre Length of the Recycled Geogrid in Relation to the Type of Mineral and Asphalt Mixture

#### 4.2.1. Utility Function Method

A multi-criteria statistical optimisation method was used to perform a comprehensive evaluation of the research object (material), taking into account the assumed evaluation criteria. The multi-criteria optimisation was realised on the basis of Harrington’s utility function method [61]. In this method, the values of all considered criteria are expressed on a common dimensionless scale. Constructing such a scale requires defining a range of satisfactory values for each criterion. In addition to the satisfaction intervals, non-negative coefficients reflecting the importance of the criteria are also assigned to the individual criteria. The sum of the weights must, of course, be equal to 1. The adoption of the satisfaction intervals and the weights for the individual characteristics is based on knowledge of the technical requirements, or having expert knowledge of the properties attributed to the material in question. The sub-utilities d_i_ are numbers in the range <0;1>, with numbers close to 0 corresponding to particularly unfavourable values of the characteristic y^(i)^, and numbers close to 1 corresponding to the most favourable values. The set of y^(i)^ values considered satisfactory, taking into account the non-linear model of the profile function, is in the interval (0.368; 0.632). Values below 0.368 should be interpreted as an unsatisfactory result, in relation to the feature in question, in light of the criteria assigned to it. In contrast, values of partial utility d_i_ > 0.632 should be interpreted as a good result. In this range of utility, the profile function is most sensitive to a change in the value of a given feature [56]. The qualitative ranges of the utility function are shown in Table 5.

Utilities d_i_ attributed to individual characteristics y(i) are determined by two algorithms depending on whether the recommended value determined from the criteria is in the middle of a certain interval (e.g., free space content) or is below or above a certain value (e.g., water resistance). Therefore, two types of profile functions have to be used, which bring the variability in a given characteristic into the interval <0;1>. Their formulae are shown below (Table 6).

Finally, the generalised utility should be determined, which will determine the resultant value of the sub-utilities. In this way, the quality of the material and the most optimal solution can be determined using a single measure. In the case of this analysis, these are the sought-after parameter values of the recycled geogrid in AC. The generalised utility *D* is determined as the geometric mean of the individual *d_i_* (13):

(13) $$D={\left[\overset{n}{\underset{i=1}{\prod }}d_{i}\right]}^{\frac{1}{n}}$$

where *di*—result of a given partial utility function; n—number of variables. This type of profiling is very sensitive to the occurrence of values close to *d_i_* = 0.37 in Equation (12). This will ensure that values close to the limits will not have a significant effect on the increase in the *D* score. On the other hand, a value of *d_i_* = 0 will disqualify the need to search for an optimum, as it will not occur in this case. Thus, this optimisation approach favours the occurrence of values that are in the middle of the two-sided function or at a significant distance from the unacceptable values in the one-sided function notation. In summary, the optimisation task will be to look for a solution or a solution area in which the utility function will take the value *D* > 0.37.

#### 4.2.2. Optimal Criteria and Solutions

The optimisation criteria were adopted by suggesting the standard requirements for asphalt concrete intended for the binder course according to WT-2/2014 (Poland) [22]. It should be borne in mind that changing at least one criterion significantly affects the result of estimating the optimum solution in terms of the material properties sought. Therefore, the main criterion on which the optimisation result depended was to obtain a solution close to the middle value of the V characteristic and at least satisfactory values for the other properties (*d_i_* > 0.37). The optimisation criteria used are presented in Table 7.

**Figure 18:**
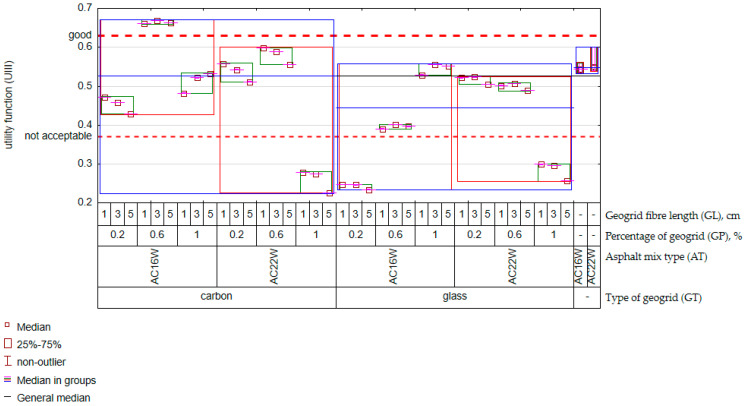
Result of optimising the parameters of the recycled geogrid.

The most important consideration was the need to provide the required stiffness as defined by the Polish Catalogue [37], ensuring low creep rates and adequate water resistance. Predicted results from the previously determined GLZ models were used for the optimisation (Table 4). In order to improve the multiple approximation process of the optimisation process, a script was prepared in R [62] and entered into Statistica. The results of the resulting response are shown in Figure 18.

For comparison purposes, the result of the assigned value of the utility function for the reference mixtures was added to Figure 18. It should be noted that the result obtained suggests that the reference mixes scored on the borderline between satisfactory and good. Referring to the evaluation of the AC containing the geogrid, it should be noted that the presence of the carbon geogrid definitely increased the quality of the asphalt concrete in terms of the utility function. When the carbon geogrid was used, the utility of the material was above average (median *D* > 0.5) compared to the glass mesh (median *D* < 0.5). In addition, more favourable results were obtained in most cases where the mixture was fine-grained (AC16W) than when a mineral skeleton with a maximum grain size of 22 mm (AC22W) was used as the matrix. Arguably, the aforementioned optimum relationship between grain size and fibre length in AC16W was important in increasing the quality of the composite. It also turned out that the glass mesh could be dosed at 1% for fibre lengths of up to 5 cm in the AC16W mix. In contrast, for the same mix, carbon fibres achieved a positive result when the amount was 0.6%. Further increases in the carbon mesh in AC16W resulted in a significant reduction in the quality of the material, which could be due to supersaturation and lack of space for further geogrid implementation. For AC22W mixes, adequate quality could be achieved when the geogrid content, either glass or carbon mesh, was <0.2%. However, for AC22W mixtures, a geogrid content above 0.6% becomes unjustifiable. It was also an interesting observation that the quality of fine-grained mixtures depended much more on the fibre length than in coarse-grained mixtures. The recommended recycled geogrid contents determined from the experiment are shown below in tabular form (Table 8).

Observing the optimisation results shown in Table 8, it should be noted that the carbon fibres in the RAP caused a positive increase in the quality of the AC16W-type mixture, proving that the amount of impurities in the form of geogrid fragments up to 5 cm long can be used up to 0.6%. It should be recalled that, according to the recommendations of WT-2/2014, the maximum amount of impurities from synthetic materials (geogrids) is 0.1%. Furthermore, in the vast majority of cases, the presence of mesh in an amount >0.1% did not result in a significant difference in the quality of the material compared to the quality achieved by the reference mixtures. Comparing the optimisation results obtained with respect to the quality of the reference samples, a significant relative increase in material quality was obtained for the case of carbon geogrid fibres:

- AC16W (GP = 0.6%, GL ∈ <1 cm; 5 cm>) by 22%,
- AC22W (GP = 0.6%, GL ∈ (1 cm; 5 cm>) by approximately 9%.

In contrast, in the case of the glass fibre mesh, the quality of the material was at a comparable level (difference < 5% compared to the reference mix) and only for the following cases:

- AC16W, GP = 1%, GL ∈ <1 cm; 5 cm>,
- AC22W, GP = 0.2%, GL ∈ <1 cm; 5 cm>.

Taking into account the survey and the results of the optimisation, it can be concluded that the introduction of an additional control in terms of the level of contamination of the RAP with geogrid fibres will allow the safe use of more contamination > 0.1%. It should be noted that the optimisation presented relates to the range of the experimental domain used. This model can be used within a small extrapolation range due to the fact that the residuals calculated in the models had a normal distribution. If other composites with different grain sizes are used, additional verification studies are required, which is the main objective of the authors. This observation pertains to the proposed Equations (11) and (12), where the parameters will be calibrated based on the number of experimental samples using machine learning techniques.

## 5. Conclusions

On the basis of the research and analysis carried out on the effect of synthetic contaminants in the form of geogrids on the properties of asphalt concrete, the following conclusions were drawn:

- The findings of the survey indicate the necessity to undertake research on the issue of RAP contamination by geosynthetic materials. The allowable maximum concentration of less than 0.1% poses a substantial restriction on the feasibility of incorporating RAP contaminated with geogrids. Conversely, this study suggests notable enhancements in the properties of asphalt concrete when RAP integrated with geogrids is utilised, given that RAP is accurately identified.
- An important issue identified by the surveys is that reclaimed asphalt pavement (RAP) is frequently contaminated with geogrid segments exceeding 5 cm in length, which are not further analysed. If these segments constitute more than 0.1% of the RAP, the material is deemed unsuitable for subsequent use. Moreover, the survey emphasized the necessity for additional research to persuade designers and construction authorities to adopt RAP with geogrid on a broader scale.
- The tests carried out confirmed the hypothesis of a beneficial effect of geogrid contamination in the form of >0.1% *w*/*w* asphalt concrete. However, a precise recognition of its geometrical properties, its distribution in the asphalt concrete, and the material from which it is made are required here.
- The carbon mesh found in the destruction effectively increases the quality of the asphalt concrete in the binder course by 22% compared to the reference solution. This effect is possible if the fibres were dosed at a rate of 0.9% m/m and a length of no more than 5 cm. An increase in the quality of asphaltic concrete for the binder course based on the utility function is possible for mixtures with a maximum grain size of 16 mm and 22 mm.
- In the case of glass mesh, its accepted content in the composition must not exceed 1% for a mix with a maximum grain size of 16 mm and 0.2% in a mineral mix with a maximum grain size of 22 mm.
- The proposed GLZ model proved to be an effective tool for aggregate prediction of physical and mechanical characteristics for different asphalt mix variants and geogrid material types.
- The choice of mesh length in the waste should be confronted with the expected grain size of the asphalt concrete mineral mixture. It is a very good solution to maintain the rule used in fibre concretes that the fibre length should not be greater than three times the maximum aggregate grain size in the mineral mixture. Then, especially in the case of carbon mesh, a significant reduction in permanent deformation (WTS_AIR_) and an increase in ITSR much greater than in the case of the AC22W mix was achieved in the AC16W mix.
- The wear process of the geogrid in the RAP varies and depends on the material used. In the case of the glass geogrid, the level of absorption was higher than that of the carbon fibre mesh, resulting in an increase in the void content of the asphalt concrete. As a result, testing of the RAP contaminated with the geosynthetic material requires additional attention in recognising its suitability.

## Figures and Tables

**Table 1:** Properties of selected geogrids.

Property	I.M.	Glass Geogrid G 100/100	Carbon–Carbon Geogrid 100/100
Breaking force—along	kN/m	≥100	≥100
Breaking force—across	kN/m	≥100	≥100
Maximum elongation—longitudinal	%	≤3	≤1.5
Maximum elongation—lateral	%	≤3	≤1.5
Mesh size—along	mm	39	40
Mesh size—wide	mm	39	40
Thermal resistance	°C	min. 265	min. 2000
Surface mass	g/m^2^	450 (±30)	95 (±7)
Coverage	-	polymeroasfalt	polymeroasfalt

**Table 2:** Input variables and their levels in the experimental plan.

Quantitative Variable	Levels	Qualitative Variable	Levels
Percentage of geogrid (GP), %	0.2; 0.6; 1.0	Type of geogrid (GT)	carbon; glass
Geogrid fibre length (GL), cm	1; 3; 5	Asphalt mix type (AT)	AC16W; AC22W

**Table 3:** Domain of experiment.

Case	Type of Geogrid (GT)	Type of Mixture (AT)	Percentage of Geogrid (GP)	Geogrid Fibre Length (GL)
1	glass	AC16W	0.2	1
2	glass	AC16W	0.6	3
3	glass	AC16W	1.0	5
4	glass	AC22W	0.2	1
5	glass	AC22W	1.0	5
6	carbon	AC16W	0.2	1
7	carbon	AC16W	0.6	3
8	carbon	AC16W	1.0	5
9	carbon	AC22W	0.2	1
10	carbon	AC22W	0.6	3
11	carbon	AC22W	1.0	5
12	carbon	AC22W	1.0	1
13	carbon	AC16W	0.2	5
14	glass	AC22W	0.2	5

**Table 4:** Parameters of the GLZ models of the analysed characteristics of asphalt concretes.

L.P.	Effect	ITSR	PRD_AIR_	Sm13	V_a_	WTS_AIR_
1.	Type of Geogrid (GT)	16.147177	NS	NS	NS	NS
2.	Type of Geogrid (GT)*Geogrid fibre length [cm] (GL)	NS	NS	NS	NS	−0.004475610
3.	Type of Geogrid (GT)*Type of MMA mixture (AT)	NS	NS	557.8295	0.6514006	NS
4.	Type of Geogrid (GT)*Share of Geogrid [%] (GP)	−19.006170	NS	NS	NS	NS
5.	Mixture type MMA (AT)	NS	0.381250	NS	−1.1005136	0.008934451
6.	Mixture type MMA (AT)*Percentage of geogrid [%] (GP)	NS	−1.031250	3349.8058	NS	−0.024184451
7.	Percentage of geogrid [%] (GP)	NS	4.468750	NS	4.4651024	0.105586890
8.	Percentage of geogrid [%]*Geogrid fibre length [cm] (GL)	3.064244	NS	−1120.2259	NS	NS
9.	Intercept	86.561718	4.778125	NS	2.8895635	0.039696
	R^2^	0.83	0.92	0.75	0.87	0.77
	RMSE	7.0	0.4	2292	1.1	0.014

NS—non-significant.

**Table 5:** Assessment of the qualitative utility function *d_i_* [56].

Quality Range	Interpretation
1.00	Excellent value.
1.00–0.83	A very good value, representing the achievement of a quality material with remarkable optimisation properties.
0.8–0.63	It represents good above average quality.
0.63–0.37	Satisfactory value, acceptable under certain conditions.
0.37–0.2	An unacceptable value, which may increase the unreliability of the optimised product.

**Table 6:** Formulae for profile functions (partial utility d_i_)

Asymmetrical Function	Symmetrical Function
$d_{i}=e^{-{\left(\left|\frac{2\cdot y_{i}-\left(y_{max}-y_{min}\right)}{y_{max}-y_{min}}\right|\right)}^{n}}$ where *y_min_*. *y_max_*—the lower *and upper* range for the criteria defined; *y_i_*—the current result; the value from the range; n—an odd integer value (n = 1 assumed)	$d_{i}=e^{-e^{-\left(\left|\frac{\left(y_{i}-y_{worse}\right)}{y_{good}-y_{worse}}\right|\right)}}$ where *y_good_*—accepted values above the cut-off value; *y_worse_*—cut-off value of results to be rejected; *y_i_*—current result

**Table 7:** Criteria adopted for optimisation based on WT-2/2014 [22].

	1	2	3	4	5
	V_a_ [%]	ITSR [%]	Sm (+13 °C) [MPa]	WTS_AIR_ [mm/1000 Cycles]	PRD_AIR_ [%]
Worse (*d_i_* < 0.37)	4.0 ÷ 7.0	0.9	10,300	0.15	7.0
Good (*d_i_* > 0.63)		0.95	14,000	0.1	5.0

Criteria applied to the recycled MCAS mix.

**Table 8:** Recommended values for the use of recycled geogrids.

Composite Quality According to Table 7	Type	Type of Fibre (Recycled Geogrid)	
		Carbon	Glass
Good, above average	AC16W	fibre length ∈ <1 cm; 5 cm> share 0.6%	none
	AC22W	none	none
Satisfactory, acceptable under certain conditions	AC16W	fibre length ≈ 1 cm share ∈ <0.2%; 0.6%>	fibre length ∈ <1 cm; 5 cm> share ∈ <0.6%; 1%>
	AC22W	fibre length ∈ (1 cm; 5 cm> share ∈ <0.6%; 1%>	fibre length ∈ <1 cm; 5 cm> share ∈ <0.2%; 0.6%>

## Data Availability

Data available on request from the corresponding author.
